# Historical review and occurrence records of *Callipogon relictus* Semenov, 1899 (Coleoptera, Cerambycidae) in Gwangneung Forest, South Korea with suggestions for species conservation

**DOI:** 10.3897/zookeys.1024.61483

**Published:** 2021-03-15

**Authors:** Seung-Gyu Lee, Bong-Woo Lee, Cheol-Hak Kim, Jung Hoon Kang, Seung-Hwan Oh, Jongok Lim

**Affiliations:** 1 Animal Resources Division, National Institute of Biological Resources, Incheon, South Korea National Institute of Biological Resources Incheon South Korea; 2 Division of Forest Biodiversity, Korea National Arboretum, Pocheon, Gyeonggi Province, South Korea Korea National Arboretum Pocheon South Korea; 3 Osang K-insect Biological Resource Research Center, Yesan, Chungnam Province, South Korea Biological Resource Research Center Yesan South Korea; 4 National Research Institute of Cultural Heritage, Daejeon, South Korea National Research Institute of Cultural Heritage Daejeon South Korea; 5 School of Forest Sciences and Landscape Architecture, Gyeongbuk National University, Daegu, South Korea Gyeongbuk National University Daegu South Korea; 6 Department of Bio-Environmental Chemistry, Wonkwang University, Iksan, Jeonbuk Province, South Korea Wonkwang University Iksan South Korea

**Keywords:** Biodiversity, endangered species, longhorn beetle, natural monument, South Korea

## Abstract

Biodiversity has been declining and extinction rates have been exponentially increasing because of land use changes, invasion of exotic species, nutrient enrichment and climate change. In this scenario, many international networks such as the International Union for the Conservation of Nature have been making efforts to raise conservation awareness and preserve species and their habitats in many countries. The relict longhorn beetle *Callipogon relictus* Semenov, 1899 (Coleoptera: Cerambycidae) is the largest coleopteran species in the Palearctic region and has a unique distribution compared to its congeneric species. *Callipogon relictus* has been protected by two Korean laws since it was designated as a Korean Natural Monument and an Endangered Species in 1968 and 2012, respectively. To improve the conservation of this species, ecological and biological data were obtained from studies performed during the last 12 years on its populations in Gwangneung Forest, the fourth UNESCO biosphere reserve in South Korea. Previously scattered distribution records of *C. relictus* from South Korea from 1932 to 2007 are therefore summarized and ecological features of adults observed during fields studies performed from 2008 to 2019 are presented. Based on the summarized data, we suggest different management measures and conservation efforts to maintain the size of *C. relictus* populations in South Korea, which can also be further used in the restoration of other endangered insects.

## Introduction

Land use changes, invasion of exotic species, nutrient enrichment and climate change are factors that have been influencing global ecosystem changes (Isbell 2010). Moreover, anthropic activities have resulted in contemporary biodiversity declines and exponentially increased extinction rates (Lugo 2006). Recent regional biomonitoring reports have indicated a multicontinental crisis of insects. Despite being fundamental to terrestrial and freshwater ecosystem processes, these animals have been suffering reductions in abundance, diversity and biomass (Forister et al. 2019).

In 2016, a total of 343 insect species were assessed as Endangered in the International Union for the Conservation of Nature (IUCN) Red list of Threatened species, among which 72 were coleopteran species (IUCN 2016). As of 2020, the IUCN Red List of Threatened species is targeting a total of 160,000 species for analysis, of which 12,400 species belong to the class Insecta (IUCN 2020). In South Korea, three insect species (*Callipogon relictus* Semenov, 1899, *Chrysochroa coreana* Han & Park, 2012 and *Hipparchia autonoe* (Esper, 1783)) and a habitation of two lampyrid species, *Aquatica lateralis* (Motschulsky, 1860) and *Lychnuris rufa* (Olivier, 1886) were designated as Korean Natural Monuments and are therefore protected by the Korean “Cultural Heritage Protection Act”. Moreover, 26 insect species have been designated as Endangered Species and are protected by the Korean “Wildlife Protection and Management Act”.

*Callipogon relictus* was first described from Vladivostok, Eastern Russia (Semenov 1899). The species belongs to the genus *Callipogon* Audinet-Serville, 1832 and its eight congeneric species are distributed in North, Central and South America including Mexico, Guatemala and Colombia (Lameere 1904; Monné 2017). *Callipogon relictus* was recorded from China, Russia and the Korean Peninsula (Byun et al. 2007; Li et al. 2013; Bezborodov 2016; Yi et al. 2018). Its disjoint distribution is considered evidence of a connection between the Old World and the New World by the Bering land-bridge (Kuprin and Bezborodov 2012; Lee et al. 2018). Kim et al. (2018) showed that *Callipogon* originated in the Paleocene ca. 60 million years ago across the Neotropics and Eastern Palearctic.

*Callipogon relictus* is protected by the South Korean government because of its rapid decreases in population density. This species was designated as a Korean Natural Monument (No. 218) on November 25, 1968 and as an Endangered Species (Critically Endangered A1[a,c]; B1, B2[a]) on December 6, 2013 (Suh et al. 2014; Lee et al. 2018). However, because of the complex ecological features that hinder population studies of this species individuals have a long lifespan (5–7 years) inside host plants (Li et al. 2012; Danilevsky 2014) and only a short adult period (28 days) (Danilevsky 2014; Kuprin et al. 2014). This species has not yet been included in the IUCN Red List.

A living *C. relictus* female was found in Gwangneung Forest in 2006. After that, the ecological characteristics of the species were studied to determine the status of the populations in the forest. In 2014, a living male was found. Since then, a total of 16 adults have been recorded from this location, with individuals being found every year (Byun et al. 2007; Lim et al. 2013, 2017; Lee et al. 2018, 2019). In 2017, a living female and eggs were collected from the location and *C. relictus* was bred in the laboratory. Then, three males and three females were released in 2018 and 2019, respectively; these individuals were the second generation (F2) of the female that was found in Gwangneung Forest in 2017. Moreover, two males that were found in 2018 and 2019 were released in each year. Based on this context, the aim of this study was to review the historical records on Korean *C. relictus* from 1932 to 2007, as well as to provide information on the ecological features of adults obtained during studies in Gwangneung Forest. Moreover, we provide management measures and a conservation manual on the maintenance of *C. relictus* populations in South Korea based on long-term data which can be further used in the restoration of other endangered insect species.

## Materials and methods

### Historical review on records of *Callipogon relictus* in South Korea

Historical data from 1932 to 2007 on distribution records and ecological features of *C. relictus* in South Korea were summarized. South Korean government policies, such as the designation of *C. relictus* as a Korean Natural Monument and Korean Endangered Species, were included in the data so that the efforts of the South Korean government regarding the conservation of *C. relictus* are considered.

### *Callipogon relictus* in Gwangneung Forest, South Korea

#### Investigation site

**Figure 1.**
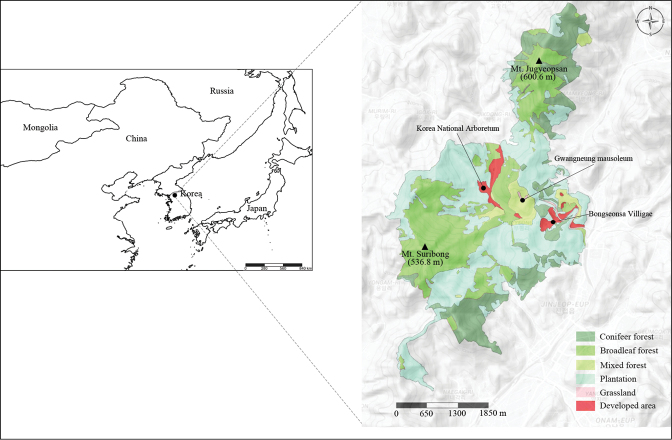
Location of the study site, Gwangneung Forest, in Korea.

The investigation site in Gwangneung Forest was near three towns, Namyangju, Pocheon and Uijeongbu in Gyeonggi province, South Korea (Fig. 1). The forest is annexed to a royal tomb, called “Gwangneung”, in which the seventh king of the Joseon Dynasty, “Sejo”, was buried approximately 600 years ago.

This forest is a cool temperate forest and it is located in the central Korean Peninsula (37°42'36"–37°47'41"N, 127°8'20"–127°11'58"E) at 40–620 m a.s.l. (Cho et al. 2007). The forest was designated as the fourth United Nations Educational, Scientific and Cultural Organization (UNESCO) biosphere reserve in South Korea because of its representative old-growth forest, which has been protected since the 15^th^ century (Cho et al. 2012; Lee et al. 2019).

#### Study period

The study was performed from June to September, which is the adult flight season. Surveys were especially concentrated from July to August, when *C. relictus* individuals were frequently found (Kim et al. 1976; Hong and Kim 1991; Byun et al. 2007; An 2010). A total of 30 surveys were performed per year during the day and at night.

#### Survey

**Figure 2.**
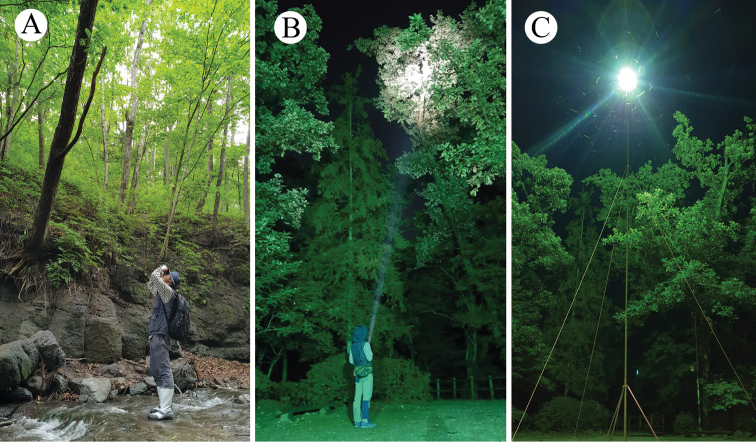
Methods used in the detection of *Callipogon relictus***A** direct search on host trees using naked eyes or binoculars **B** search using lantern **C** light resource on the 10-m tree top.

For daytime surveys, binoculars and naked-eye detection were used in the search for adults near the sap of host trees (Fig. 2A, B); this was based on previous reports of *C. relictus* feeding on sap and ferment fluid from trunks of wood species and rotten oak tree bark (Cherepanov 1988; Hong and Kim 1991; An 2010; Li et al. 2012; Danilevsky 2014; Kuprin et al. 2014). For night-time surveys, lanterns and light traps (1,000 W, 10 m) were used to attract adults (Fig. 2C) based on the suggestions of previous studies (e.g., Lingafelter (2015) and Santos-Silva et al. (2018) for giant prionine cerambycids and Byun et al. (2007) and Danilevsky (2014) for *C. relictus*). For all *C. relictus* individuals found, body length and condition (including if they were dead or a living) were recorded. Information on *C. relictus* individuals found from 2014 to 2019 are summarized in the present study. However, the exact localities of these *C. relictus* individuals are not presented in the present paper to ensure the preservation of the Gwangneung Forest habitats.

#### Breeding

**Figure 3.**
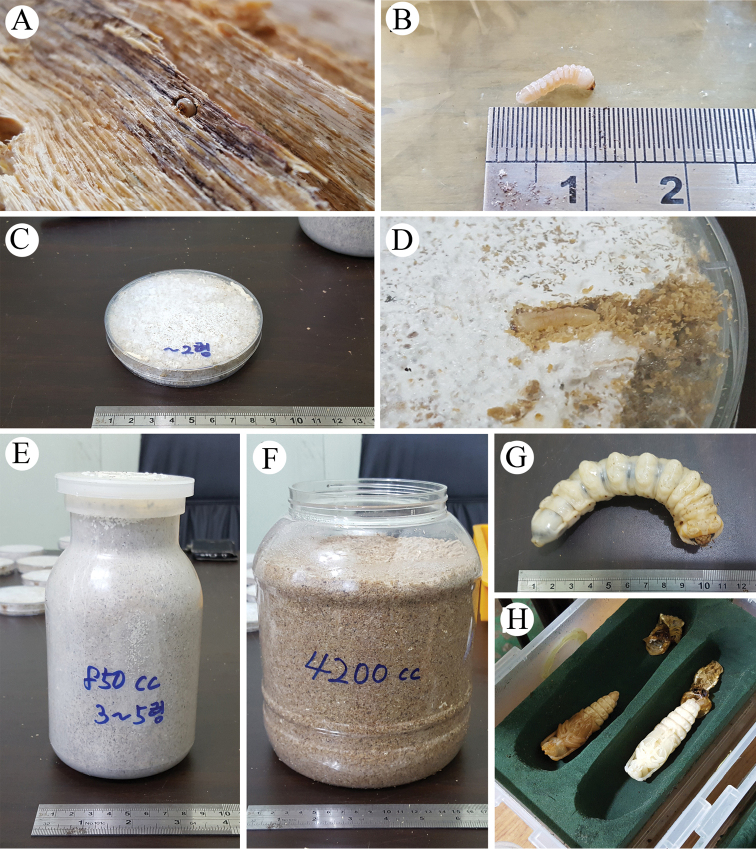
*Callipogon relictus* breeding process under laboratory conditions **A** hatched larva **B** body length of a hatched larva **C** petri dish for breeding of instars 1–2 **D** feeding of larva in a petri dish **E** 850 ml bottle for breeding of instars 3–5 **F** 4200-ml bottle for breeding of instars 5–8 **G** matured larva **H** pupae.

In 2017, one living female was collected and 16 eggs were obtained from that female (Fig. 3A, B, D). With this, breeding of Korean *C. relictus* was performed for the first time to obtain individuals for restoration or to release into the origin habitat. Each individual of larvae and pupae was separately reared in a petri dish (instars 1–2^th^; Fig. 3C), 850 ml bottle (instars 3–5^th^; Fig. 3E), or 4200 ml bottle (instars 6–8^th^; Fig. 3F, G) at 20±5 °C and a humidity of 35±1%. Pre-pupae were moved to plastic containers for adult molting (Fig. 3H). The second generation (three males and three females) of the living female found in 2017 were released in the study area in 2018.

#### Release and observation of the behaviors of adults

Two adult males and three adult females that were the second generation of the female collected on July 20, 2017 were released on July 10, 2018 and August 16, 2018, respectively. After the release of each of these adults, behaviors such as flight and tree climbing behavior were observed and compared between daytime and night-time.

## Results

### Historical review of *Callipogon relictus* in South Korea

The distribution records of the species in South Korea from 1932 to 2007 are summarized in Table 1.

**Table 1.** Summary of the historical notes on *Callipogon relictus* Semenov in Korea (1932–2019) (*, Cultural Heritage Administration).

Reference	Main contents
Saito (1932)	– First record of *C. relictus* in Korea as “*Macrotoma fisheri* Waterhouse?”
Cho (1934)	– Historical review of 13 Korean cerambycids
	– Replacement of *Macrotoma fisheri* with *Callipogon relictus*
	– New distribution record: Namjangdae in Mt. Bukhan (Seoul).
Murayama (1936)	– First record of a host plant, *Carpinus laxiflora* from Gwangneung Forest
	– Description of larvae and presentation of biometric data for body parts.
Cho (1946)	– Checklist of 205 Korean cerambycids with synonyms and distribution range
	– Summary of distribution records of *C. relictus* in Korea: Chujeon-ri, Chuncheon; Georye-ri and Yuchon-ri, Hwacheon; Yanggu-eub, Yanggu; Mt. Bukhan, Seoul.
Cho (1959)	– Presentation of the records of host plants of Korean cerambycid species
	– Records of two host plants, *Quercus mongolica* and *Carpinus laxiflora*
	– Replacement of the Korean common name for cerambycids “Ha-nul-so” with “Ha-neul-so”.
Cho (1961)	– First comprehensive taxonomic study on 217 Korean cerambycids
	– Description of the sexual dimorphism of *C. relictus* and distribution range of the species in Korea.
CHA* (1962)	– Designation of habitat, “Chujeon-ri, Buksan-myeon, Chuncheon-si”, as a Korean Natural Monument (No. 75).
CHA* (1968)	– Designation of *C. relictus* as a Korean Natural Monument (No. 218).
Kim and Kim (1971)	– New distribution record of *C. relictus*: Cheokhak-dong, Sogeumgang (Gangwon Province).
CHA* (1973)	– Cancellation of the designation of “Chujeon-ri, Buksan-myeon, Chuncheon-si” as a Korean Natural Monument (No. 75) because of habitat loss.
Kim et al. (1974)	– Publication of “Distribution Atlas of Insects of Korea 1. Family Cerambycidae”
	– Presentation of *C. relictus* as “Callipogon (Callipogon) relictus (Semenov)”.
Kim et al. (1976)	– Publication of the results of a survey of *C. relictus* from Gwangneung and Sogeumgang.
Kim (1977)	– Publication of “Distribution Atlas of Insects of Korea. Series II. Coleoptera”
	– Records of five *C. relictus* host plant taxa from Korea, China, and Russia
	– New distribution record of *C. relictus*: Cheongpyeong-ri, Buksan-myeon (Gangwon Province).
Lee (1982)	– Presentation of checklist of 261 Korean cerambycids with photographs of *C. relictus* habitus.
Kim and Nam (1982)	– Announcement of the extinction of *C. relictus* in the Seoul region.
Lee (1987)	– Publication of a book listing 292 Korean cerambycid species with synonyms, distribution range, and pictures of each species, including *C. relictus* collected from Gwangneung Forest.
Hong and Kim (1991)	– Report on the morphological variations and ecological notes with the examination of 28 individuals.
Shin (1993)	– Publication of an illustrated book on Korean insects including pictures of two individuals and a living female of *C. relictus* collected from Gwangneung Forest.
Nam (1996)	– Publication of an illustrated book on Korean insects including picture of a living female *C. relictus* collected from Gwangneung Forest.
Byun et al. (2007)	– Report on the ecological features of a female found in 2006
	– Presentation of methods and efforts for the preservation of *C. relictus* in Korea
Jang et al. (2015)	– Publication of a book of 357 Korean cerambycid species with a brief morphological characteristics and ecological features in detail including pictures of specimens and individuals in nature.
	– Introduction of *C. relictus* with pictures of Korean individuals.
Hwang (2015)	– Publication of a book of 255 Korean cerambycid species with brief morphological and ecological features including pictures of specimens and individuals in nature.
	– Introduction of *C. relictus* with pictures of Chinese specimens and individuals in breeding.
Lee et al. (2018)	– Report of the results of an investigation of *C. relictus* in Gwangneung Forest from 2007–2017
	– Comparison of results from Gwangneung Forest, Korea and Ussuri Nature Reserve, Russia
	– New distribution record: Yangyang-gun, Gangwon Province.
Lee et al. (2019)	– New host record of *C. relictus*: *Quercus aliena* Blume
	– Summary of host species (17 species of 11 genera in seven families) of *C. relictus* from Korea, China, and Russia.

*Callipogon relictus* was first recorded from the Korean peninsula by Saito (1932) as “Macrotoma (Bandar) fisheri, Waterhouse?”. In this paper, the author pointed out that only one individual was collected by Mr. Cho without information on the date of collection or habitat of the specimen.

Cho (1934) reviewed the records of Korean cerambycids from 1886 to 1933, which contained descriptions of 13 species including *C. relictus*. This author identified Saito’s (1932)*Macrotoma fisheri* as *C. relictus*. He also included information on the distribution (Namjangdae, Mt. Bukhan, Seoul) and collection dates (female, August 25, 1933; male, August 1932) of *C. relictus*.

Murayama (1936) reported *Carpinus laxiflora* Blume (Betulaceae) as a host species of *C. relictus* from Gwangneung Forest. This author also described the larval morphology and biometric measurement data for each body segment (whole body, head, prothorax, mesothorax, metathorax, abdominal segments and leg). In addition, the author indicated *Quercus mongolica*, *Fraxinus mandshurica* and *F. rhynchophylla* as host plants of *C. relictus* from Russia. Cho (1946) listed 205 Korean cerambycids and presented synonyms and the worldwide and Korean distribution for each species. The author also presented the following distribution for *C. relictus* in South Korea: Chujeon-ri, Buksan-myeon in Chuncheon-gun (Gangwon province), Georye-ri, Hanam-myeon in Hwacheon-gun (Gangwon Province), Yuchon-ri, Gandong-myeon in Hwacheon-gun (Gangwon Province), Yanggu-eub in Yanggu-gun (Gangwon Province), Mt. Bukhan (Seoul) and Gwangneung (Gyeonggi province)”. Moreover, the author suggested the following common Japanese name for *C. relictus*: Usuri-oho-kamikiri. Cho (1959) reported a survey on host plants of Korean cerambycids, in which *Quercus mongolica* and *Carpinus laxiflora* were indicated as hosts of *C. relictus*. Moreover, the author standardized the Korean name of cerambycids from “Ha-nul-so” to “Ha-neul-so”. Cho (1961) published the first comprehensive taxonomic study on 217 Korean cerambycids from 100 genera, including synonyms and distribution of each species including *C. relictus*. This study also described the sexual dimorphism of *C. relictus* and its distribution in South Korea following Cho (1946).

On December 3, 1962, “Chujeon-ri, Buksan-myeon in Chuncheon-gun” (305,910 m^2^), one of the habitats of *C. relictus*, was designated as a Korean Natural Monument (“Habitat of *Callipogon relictus* in Chuncheon” [No. 75]) by the Cultural Heritage Administration of South Korea.

On November 25, 1968, the Cultural Heritage Administration of South Korea designated *Callipogon relictus* as a Korean Natural Monument (No. 218) because of its cultural rarity and academic significance.

Kim and Kim (1971) present a report on the insect fauna of Sogeumgang and Mt. Odae, Gangwon Province. This report includes the first record of a *C. relictus* female, which was collected from Cheonghak-dong in Sogeumgang, Gangneung-si, Gangwon Province by students of Baejae High School on July 28, 1971.

On August 14, 1973, the “Habitat of *C. relictus* in Chuncheon”, which had been designated as Korean Natural Monument No. 75, was canceled because of habitat destruction resulting from the construction of a dam.

Kim et al. (1974) published “Distribution Atlas of Insects of Korea 1. Family Cerambycidae”, with 62 species including *C. relictus*. In this paper, *C. relictus* was presented as “Callipogon (Callipogon) relictus (Semenov)” and the number of specimens deposited in two universities, two institutions and one high school was provided.

Kim et al. (1976) examined specimens that were deposited in two universities, two institutions and one high school and presented data on habitats and habits of *C. relictus* in Gwangneung, Gyeonggi province and Sogeumgang, Gangwon Province. These authors suggested that *C. relictus* was distributed only in Gwangneung Forest, Sogeumgang and that this species was living in hard trunks of *Carpinus laxiflora* of 80–250 cm in diameter, their eggs are 2.60 mm in width and 6.72 mm in length and the total larval period is 3–5 years.

Kim (1977) published the book “Distribution Atlas of Insects of Korea. Series II. Coleoptera”, with the diagnosis and collection information of 458 species of 36 coleopteran families from South and North Korea. For *C. relictus*, the author presented the following host plants: *Quercus mongolica* and *Fraxinus rhynchophylla* in South Korea, *F. mandshurica* in China, *Quercus* sp. and *Ulmus* sp. in Russia. The distribution map presented for *C. relictus* was similar to that of Kim et al. (1974), except for one new distribution record from “Cheongpyeong-ri, Buksan-myeon, Gangwon Province”.

Lee (1982) presented a checklist of 261 Korean cerambycids and provided photographs of two males collected from Gwangneung Forest on August 3 and 11, 1967. In that same year, Kim and Nam (1982) mentioned that *C. relictus*, which was previously reported from Mt. Bukhan, was not found in their survey of insect fauna in Seoul.

In the book “The Longicorn Beetles of the Korean Peninsula” Lee (1987), the author revised a previously published checklist of Korean Cerambycidae (Lee 1982). This book listed 292 Korean cerambycid species with synonyms, distribution and photographs for each species. The photographs of *C. relictus* consisted of a male with same data as that in Lee (1982) and a female collected on August 12, 1972 from Gwangneung Forest.

Hong and Kim (1991) reported on the morphological variations and ecological notes of 28 adults. According to these authors, the male mandible is different from that of other male cerambycids regarding its shape, which can be separated into long, median and short. They also commented that adults were found attached to sap bleeding from *Quercus mongolica* and that they may drop to the ground during an attack by natural enemies or fighting between individuals. They also commented that *C. relictus* flies at ~100 m aboveground and the movement of the hind wings is very loud during flight.

Shin (1993) published an illustrated book of Korean insects with photographs of adults found on July 25, 1956 and a living female found on July 7, 1987, all from Gwangneung Forest.

Nam (1996) published an illustrated book of Korean insects with photographs of a living female found on July 20, 1987 in Gwangneung Forest.

Byun and Cho (2006) and Byun et al. (2007) reported 12 records of *C. relictus* from Gwangneung Forest from 19 years of observations (1978–2006) including that of a female collected on August 24, 2006. They also proposed conservation measures for the species. Park (2007) carried out a detailed survey of *C. relictus* in Mt. Odae, Gangwon Province and suggested areas where the species is likely to be found in Mt. Odae by comparing the climate and vegetation of this region with those of Gwangneung Forest. The author also reorganized 40 Korean individuals formally registered with the Cultural Heritage Administration of South Korea, presenting their collecting sites and depository information. Nowadays, all Korean individuals must be registered and managed by the Cultural Heritage Administration because of the status as a Korean Natural Monument. In the present study, the collection sites and depository information of 48 individuals registered by the Cultural Heritage Administration (Table 3) and of 16 additional individuals collected in 2014–2019 are presented.

### Records of *C. relictus* from previous and recent (2014–2019) studies in Gwangneung Forest

**Figure 4.**
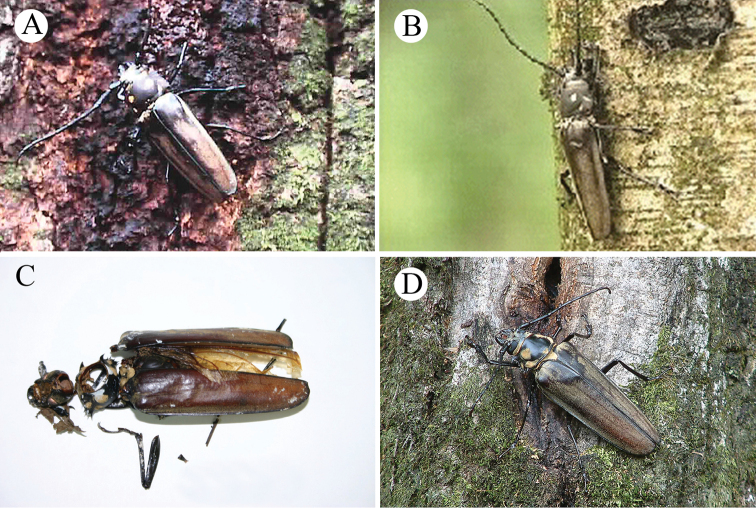
*Callipogon relictus* found in Gwangneung Forest **A** living male found in 1999, dorsal view **B** living male found in 1999, lateral view **C** body fragments of female found in 2003 **D** living female found in 2006.

**Figure 5.**
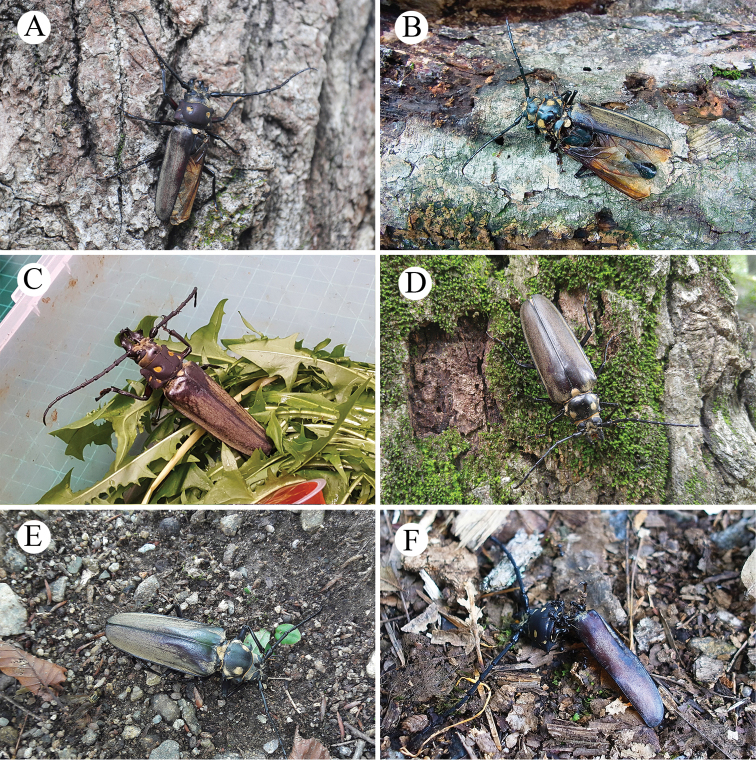
*Callipogon relictus* found in 2014–2017 in Gwangneung Forest **A** living male found on August 19, 2014 **B** dead female found on July 27, 2015 **C** living male found on August 10, 2016 **D** living female found on July 20, 2017 **E** dead female found on August 11, 2017 **F** dead male found on August 14, 2017.

**Figure 6.**
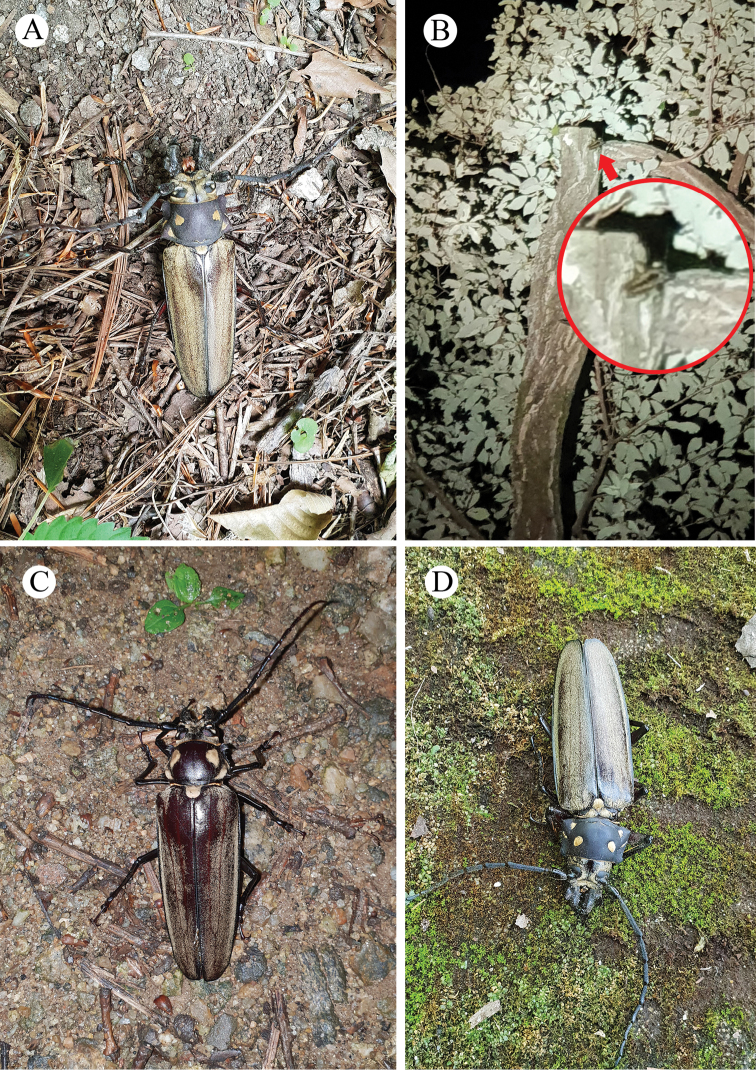
*Callipogon relictus* found in 2018 in Gwangneung Forest **A** living male found on August 6 **B** living male found on the top of a *Quercus* tree on August 13 **C** living female found on August 14 **D** dead male found on August 22.

**Figure 7.**
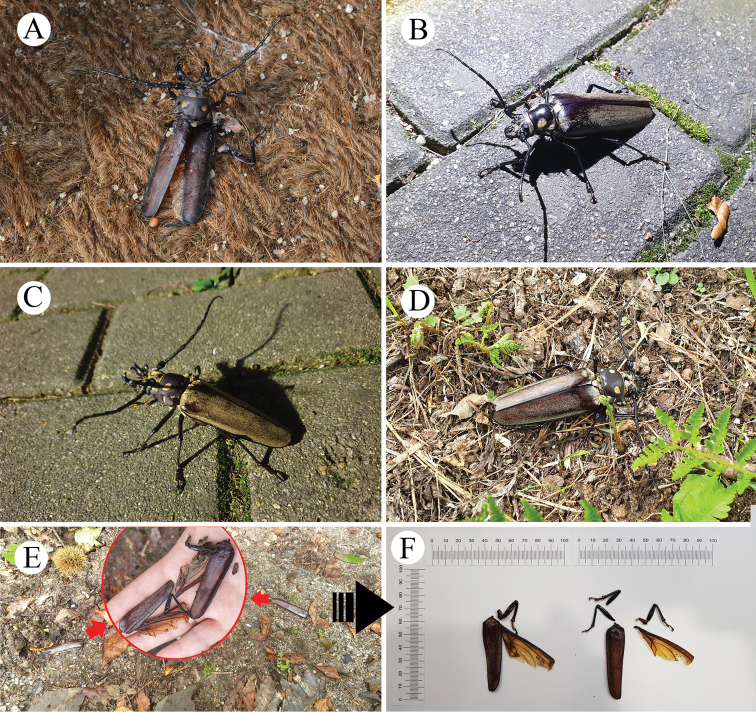
*Callipogon relictus* found in 2019 in Gwangneung Forest **A** dead male found on August 1 **B** living female found on August 6 **C** living male found on August 6 **D** dead male found on August 28 **E, F** body particles found on the ground of the forest.

Photographs of *C. relictus* from Gwangneung Forest taken from 1978 to 2019 are herein presented (Figs 4A–C, 5A–F, 6A–D, 7A–F). A living female was found in 2006 (Fig. 4D), 20 years after the first living female found in 1987. Then, a living male was collected in 2014. Since then, individuals were found every year for six years (2014–2019), totaling 16 adults from Gwangneung Forest, South Korea (Table 2). Descriptions of these individuals found in 2014–2019 are provided herein.

**Table 2.** Month and date of *Callipogon relictus* Semenov detection from 2014–2019 in Gwangneung Forest, Korea.

		**2014**	**2015**	**2016**	**2017**	**2018**	**2019**	**Total**
July **(2**♀)	20				♀			**1**♀
	21							
	22							
	23							
	24							
	25							
	26							
	27		♀					**1**♀
	28							
	29							
	30							
	31							
August (**10**♂, **4**♀)	1						♂	**1**♂
	2							
	3							
	4							
	5							
	6					♂	♂, ♀	**2**♂, **1**♀
	7							
	8							
	9							
	10			♂				**1**♂
	11				♀			**1**♀
	12							
	13					♂		**1**♂
	14				♂	♀		**1**♂, **1**♀
	15							
	16							
	17							
	18							
	19	♂						
	20							
	21							
	22					♂		**1**♂
	23							
	24							
	25							
	26							
	27						♂, ♀	**1**♂, **1**♀
	28						♂	**1**♂
**Total**		**1**♂	**1**♀	**1**♂	**1**♂, **2**♀	**3**♂, **1**♀	**4**♂, **2**♀	**10**♂, **6**♀

**Table 3.** *Callipogon relictus* specimens registered by the Cultural Heritage Administration of Korea until 2019 (*: **GF**, Gwangneung Forest; **SGG**, Sogeumgang).

Depository		No. specimen	Collection locality*
Institution	Korea Nat’l Arboretum	6 (2♂, 4♀)	GF
	National Research Institute of Cultural Heritage	14 (8♂, 5♀, 1 larva)	GF
University (**Univ.**)	Kyung Hee Univ.	6 (4♂, 2♀)	GF
	Ewha Womans Univ.	2 (1♂, 1♀)	
High School (**HS**)	Jeongeui Girls’ HS	1 (1♀)	GF
	Paichai HS	3 (1♂, 2♀)	GF, SGG
	Namkang HS	2 (1♂, 1♀)	GF
	Paiwha Girls’ HS	3 (1♂, 2♀)	GF
Private collection	G.J. Weon	6 (2♂, 4♀)	GF
	S.S. Kim	1 (1♂)	GF
	K.C. Sohn	2 (2♂)	GF
	S.H. Oh	2 (1♂, 1♀)	GF, SGG
**Total**		**48 (24**♂, **23**♀, **1 larva)**	

The living male collected from Gwangneung Forest on August 19, 2014 (Fig. 5A) was found under *Quercus* sp. (diameter at breast height [DBH] = 95 cm). Its body length was 88.0 mm and its right elytron and right leg were lost. When the male was detected, it was unable to climb a tree and it died the following day.

A dead female was found on July 27, 2015 (Fig. 5B). Its body length was 78.0 mm, the left elytron was lost, its head, prothorax and mesothorax were almost separated from each other and its pronotum was longitudinally broken. When the female was detected, many ants were inside the body feeding on the muscles inside the thorax and abdomen. The female died a few hours later.

On August 10, 2016, a living male was found (Fig. 5C) in front of a factory near Mt. Jukyeop, one of the peaks of Gwangneung Forest. The body length was 98.0 mm, its pronotum had a longitudinal crack medially and the pro- and mesoclaws of its left leg were lost. The male was living but exhausted when it was first detected and it died the next day. It was reported to have been attracted to the light installed in front of the factory and was attacked by a cat (*pers. comm.*).

In 2017, one living female and two dead adults (one male and one female) were found in Gwangneung Forest (Fig. 5E, F). The living female was found on July 20 and its body length was 78.0 mm (Fig. 5D). At night (22:30), it dropped from a *Quercus aliena* Blume (DBH 49 cm) in response to lantern light. When it was first found, it moved very actively and was undamaged except for the loss of pubescence on the elytra. A dead female was found under *Q. aliena* (DBH 57 cm) on August 11, with a body length of 75.6 mm (Fig. 5E); it was missing its right tibia and tarsi, had lost most of the pubescence on the anterior part of its elytra and its ovipositor was externally exposed. A dead male was found under *Q. aliena* (DBH 64 cm) on August 14 (Fig. 5F) and it had body length of 60.0 mm, the smallest among the males found to date. Its left elytron and some legs (left mid- and hindleg, right hindleg) were missing and its abdomen contained many *Lasius* ants (Hymenoptera: Formicidae), especially inside the pronotum.

In 2018, two living males, one dead male and one living female were found and collected (Fig. 6A–D). The first living male (body length = 98.0 mm) was found on August 6 under *Quercus serrata* Murray (DBH 57 cm) (Fig. 6A); when it was first found (10:52), it was upside-down but undamaged and very active. The other living male was found on August 13 moving on a branch of *Q. serrata* (Fagaceae) (DBH 47 cm) ~20 m aboveground at night (20:44) (Fig. 6B); because of the height, this individual was not collected, but its large body size and shape with robust antenna allowed its identification as *C. relictus*. A living female was then collected at night (20:35) on August 14 using a light trap installed under *Quercus aliena* (DBH 49 cm) (Fig. 6C); it dropped from the tree on to the ground around the white screen. Its body length was 88.0 mm, the largest body size among the females found to date. On August 22, a dead male was found under *Juglans mandshurica* Maxim. (Juglandaceae) (DBH 30 cm) (Fig. 6D), with a body length of 96.0 mm. When the male was first found, its body was not dry or stiff and although it was attacked by ants, it was still undamaged.

In 2019, a living male and female, two dead males and some body parts were found (Fig. 7A–F). A severely damaged male attacked by birds was found on the morning (08:07) of August 1 (Fig. 7A). Its body length was 98.0 mm. It had lost its abdomen and hindlegs and died a few hours after it was found. On August 6, a living male and female were collected near a light trap at night (female, at 20:37; male, at 21:50) (Fig. 7B, C). They were not directly attached to the white screen, but the female was found ~5 m from the screen and the male ~15 m from the screen. The body lengths of the male and female were 88.0 mm and 72.0 mm, respectively and they were both undamaged and very active. On August 27, several body parts of adults were found scattered within a radius of 1 m under *Quercus aliena* (DBH 66 cm) (Fig. 7E, F). These parts consisted of a right elytron (male, 57.0 mm), a left elytron (female, 54.0 mm), two right hind wings, a right foreleg, two right midlegs and one left hindleg. These parts were considered to be of a male and a female based on the two hind wings, the two midlegs and other morphological features that indicate sexual dimorphism. Two days later, on August 28, a dead male was found under *Quercus aliena* (DBH 105 cm) (Fig. 7D), with a body length of ~90 mm and some damage to the body. Its apical tarsus, a claw of the right midleg and a claw of the left midleg were lost and the apex of the left elytron was broken and lost. However, it was neither decomposed nor stiff when it was found.

### Behavioral characteristics

#### Pseudo-oviposition behavior

**Figure 8.**
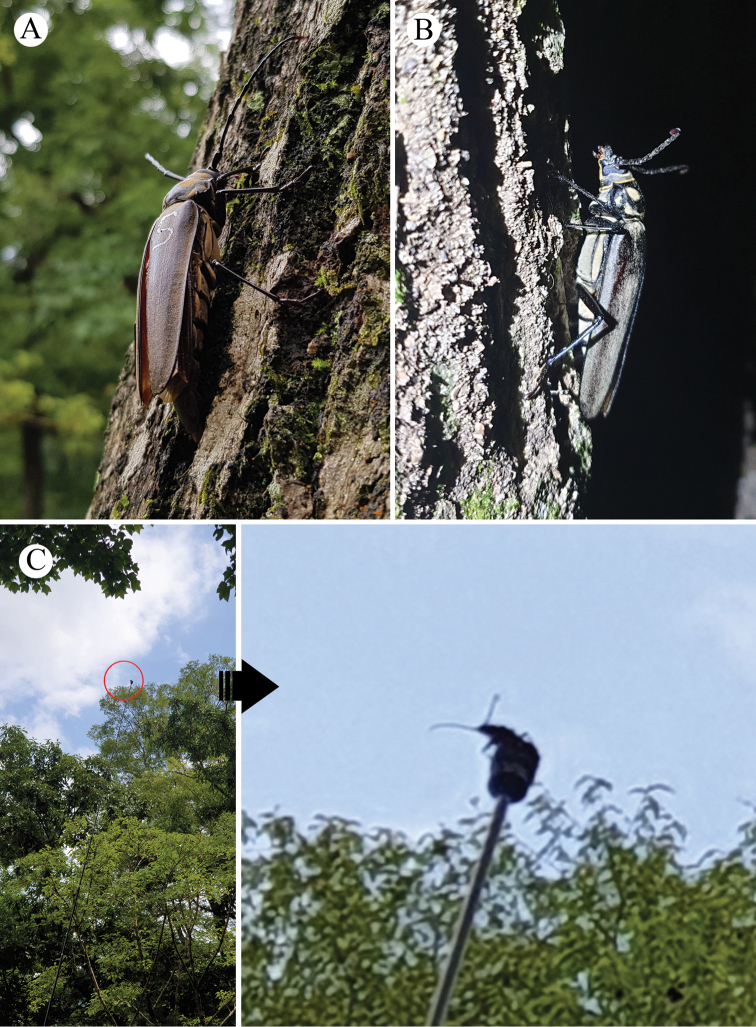
*Callipogon relictus* adults released in Gwangneung Forest **A** female released during the daytime **B** female released at night **C, D** female released from a 10-m height.

Three non-fertile females were released on *Quercus* sp. and *Carpinus laxiflora*. These specimens explored the tree bark with their antennae and tried to insert the ovipositor into cracks (oviposition behavior); however, no eggs were found in the cracks after the females were removed (Fig. 8A).

#### Climbing behavior

**Figure 9.**
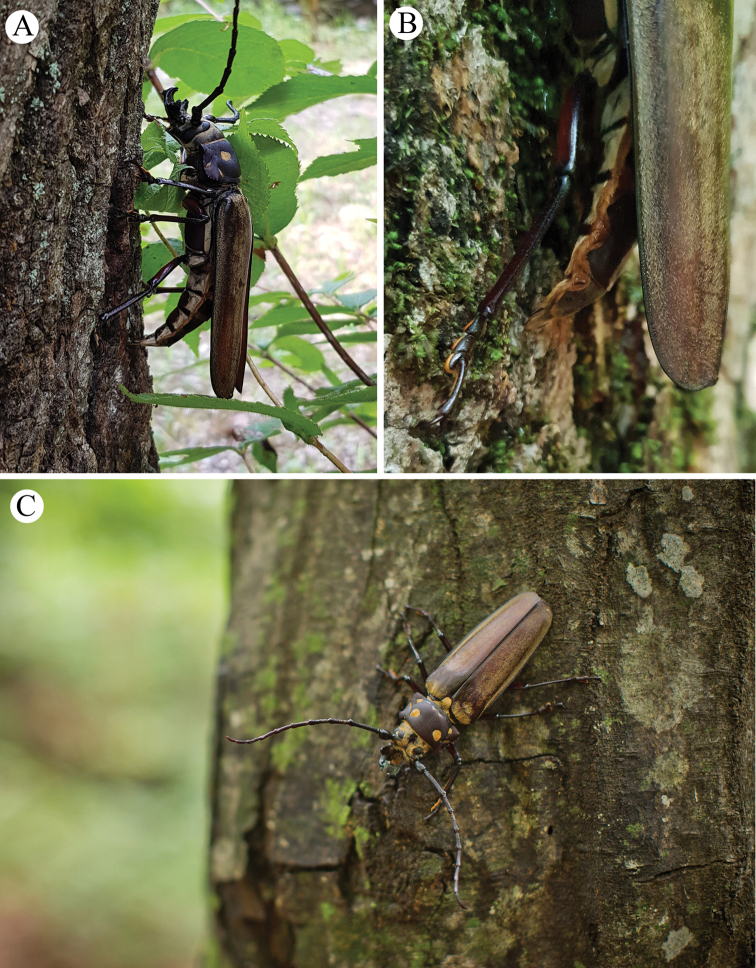
*Callipogon relictus* individuals released in Gwangneung Forest **A, B** climbing behavior of a *Callipogon relictus* male showing the C-shape curved tip of its abdomen **C** male released on the surface of a host tree.

*Callipogon relictus* was observed to use the pro- and mesoclaws to climb trees while the metaclaws supported the body. When the claws slipped on smooth bark or trees covered with water and moss, the individual would bend the abdomen downward and the apex hung from the bark in a “C”-shape to support the heavy body and prevent the animal from falling, allowing it to climb the tree (Fig. 9A, B). The individuals tended to climb to the top of the tree or walk on branches.

#### Flight behavior

**Figure 10.**
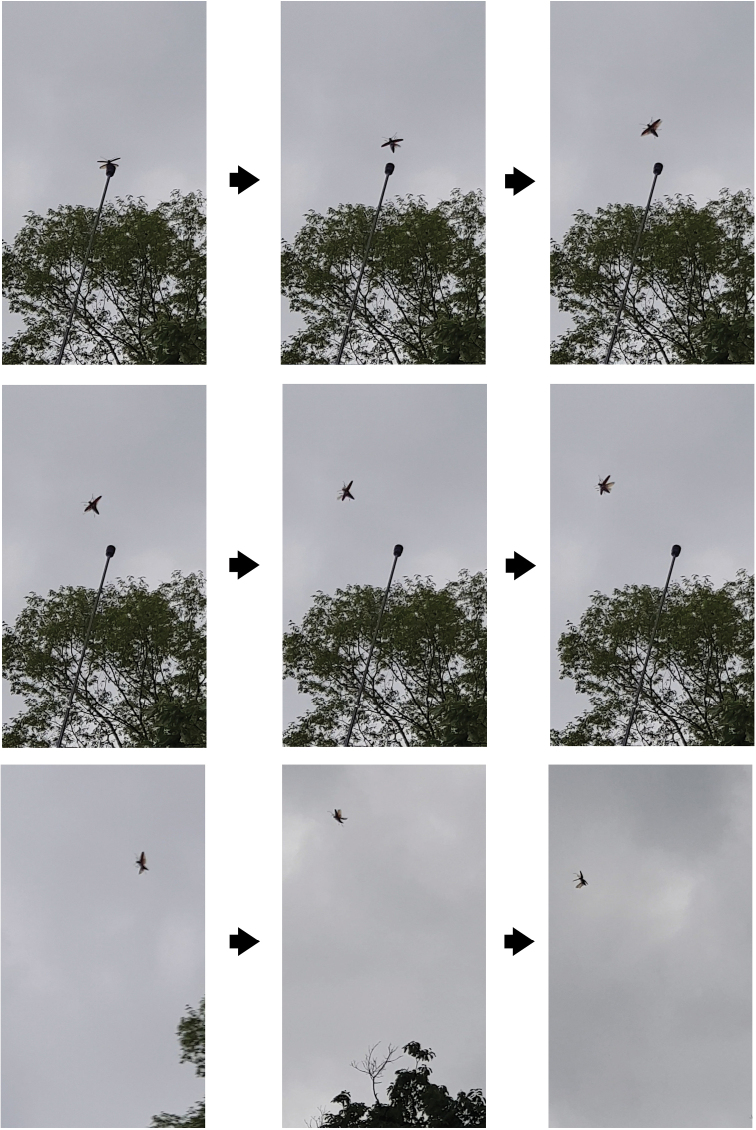
Snapshots of the flight of a *Callipogon relictus* individual from a 10-m height.

The species flight ability was relatively very good. The released adults climbed to the upper part of trees and flew for a long time (Figs 8C, D; 10). They were also able to fly downward and upward, besides flying upward from a host branch 3–4 m above ground when no obstacles surrounded them. The longest non-stop flight distance was over 500 m (the exact distance was not measured because the individual disappeared from sight). A female briefly flew beside a tree and then again to a nearby hill, relatively far from the release point.

#### Comparison of behavior during day and night-time

In the daytime, released individuals did not move for a long time, but they were vigilant about their surroundings (Fig. 8A). Then, they climbed to branches in the upper parts of a tree and hid in shady areas. A long pole (~10 m) was used to induce the flight of adults, but ~10 min were needed before flight (Figs 8C, D; 10).

Adults released at night were much less vigilant than those released during the day (Fig. 9C). As the released individuals were very active, quickly climbing to the upper parts of the tree, they were not observed for long (Fig. 8B).

## Discussion

### *Callipogon relictus* population in Gwangneung Forest

According to Byun et al. (2007), the designation of *C. relictus* as a Korean Natural Monument in 1968 indirectly implied that the population size in the area was already small and that the decline in the number of individuals was still in progress.

As Gwangneung Forest is considered the optimal habitat of *C. relictus* in South Korea, an investigation on their species inhabitation status was continuously conducted in this location for a long period. Efforts such as weekly surveys from June to September in 1970, an intensive survey conducted by six researchers for six days in 1999 and surveys conducted over 30 times by a team of 12 experts from 2005 to 2006 found no *C. relictus* adults, indicating the low population density in Gwangneung Forest (Kim et al. 1976; Byun et al. 2007). However, recent yearly surveys (2014–2019) found four individuals/100 ha and six individuals/150 ha in intensive surveys. Therefore, the current population of the species in the area seems to be relatively stable.

Previous studies reported the flight ability of *C. relictus* is poor. Yi (2014) mentioned that heavy species such as *C. relictus* use only their hind wings for descending purposes and are thus unable to perform long distance flight. However, in the present study, excellent flight ability and mobility were observed. Still, the fact that the individuals were found at the edge of the forest and that the area of Gwangneung Forest is not large enough to properly serve as a habitat for this species compared to the movement range of *C. relictus* must be considered. For stable population maintenance, this species requires an area larger than that of Gwangneung Forest and with a different distribution of host plants.

Previous studies (Kim et al. 1976; Hong and Kim 1991; Byun et al. 2007; An 2010) reported that most *C. relictus* adults in South Korea were found in August. Lee et al. (2018) also mentioned this, but commented on the fact that the state of these individuals (i.e., dead or damaged) may indicate that the period of highest activity of adults would be in July. Moreover, it should be noted that the activity period slightly varies each year depending on factors such as average temperature and precipitation and that adults with late emergence are active until the end of August.

The species is usually found around the sap of oaks (*Quercus* spp.) or on the upper parts of the trees, near food sources or oviposition sites. Individuals that fell on the ground after competition with other large beetles, e.g. lucanids and *Trypoxylus dichotoma* (Linnaeus, 1771), or attacked by natural enemies (birds, mainly woodpeckers) were often found under oaks (*Q. aliena* and *Q. serrata*). After the individuals suffered severe injuries resulting from these interspecific encounters, especially because of their large size, they are unable to hide themselves; this exposure makes these individuals vulnerable to attacks, resulting in their eventual death.

### Suggestions for the conservation of *C. relictus* in South Korea

*Callipogon relictus* adults and larvae are relatively large compared to other insects and they are popular with the South Korean public because of their strong appearance and large body. However, this results in the species being overcaught by people, which adds to the inability to quickly adapt to environmental changes, leading to its rapid population decrease. As it is legally protected in South Korea, various proposals have been made regarding the conservation of *C. relictus*. Nevertheless, although *C. relictus* has been protected as a Korean Natural Monument and an Endangered Species in South Korea, systematic management is necessary for the conservation of this species.

### Sustainable investigations of *C. relictus* occurrence and habitats in South Korea

*Callipogon relictus* is recorded from East Asia and high-density populations have been reported in forest regions in North Korea and in border regions between China and North Korea (such as Mt. Baekdu) (Kuprin and Bezborodov 2012). Collection records show that, in South Korea, *C. relictus* has been collected in Seoul (Mt. Bukhan), Pocheon (Gwangneung Forest) in Gyeonggi Province and Hwacheon, Chuncheon, Yanggu and Gangneung (Sogeumgang) in Gangwon Province. Since the 1980s, the species has been found continuously in Pocheon (Gwangneung Forest) in Gyeonggi Province. However, new population was recently detected in Yangyang-gun (Micheongol Natural Recreation Forest) (Lee et al. 2018).

Gwangneung Forest is well known as the region where *C. relictus* is most frequently found in South Korea. Therefore, investigations have been conducted in the area continuously since 1999. However, because of its low population density, nocturnal habits and preference for the upper parts of trees, the detection of individuals is difficult. In this context, although some individuals have been found every year since 2014 (Byun et al. 2007; Lee et al. 2018), the development of systematic and quantitative monitoring methods is necessary for further studies on population density. GPS tracking systems can be used for obtaining behavioral data such as flight distance and range of activity of re-introduced or released individuals. This type of data can be applied, for instance, to research on changes in population size and density. From the results of these studies, conservation measures for the populations and their habitats could be established. Nevertheless, for conservation measures to be put into practice, all actions, including collection, rearing and releasing of *C. relictus*, should require permission from the government based on the “Cultural Heritage Protection Act”, which is a process that includes several administrative steps. In this paper, we present a manual explaining in detail the process for the restoration of *C. relictus* in Gwangneung Forest, South Korea.

Gwangneung Forest is currently known as the main *C. relictus* habitat in South Korea. However, the forest areas in Gangwon Province, which have vegetation and climate similar to that of Gwangneung Forest, and the military protected areas adjacent to the DMZ, should not be disregarded as potential habitats for *C. relictus*. Kim et al. (2018) also mentioned the possibility that *C. relictus* inhabits the areas surrounding the northern border of South Korea, such as Gyeonggi Province or Taebaeksan Mountain in Gangwon Province. Lee et al. (2018) also confirmed the presence of *C. relictus* in Micheongol, Seomyeon and Yangyang-gun in Gangwon Province. Therefore, professional monitoring and sustainable and long-term investigation of *C. relictus* occurrence by cooperating agencies is necessary in areas other than Gwangneung Forest.

### Conservation of Gwangneung Forest and exploration of replacement habitats

Gwangneung Forest was designated as a UNESCO biosphere reserve (2010), being the fourth area to be designated as such in South Korea after Seoraksan (1982), Jeju Island (2002) and Sinan (Dadohae) (2009). Only 24,465 ha of Gwangneung Forest was designated as a UNESCO biosphere reserve, comprising ~2.4% of the countries land (total area 10,033,949 ha) but approximately 23% of the insect species recorded in South Korea (Byun and Cho 2006). Moreover, seven Korean endangered insect species, including *C. relictus*, inhabit this forest and besides having the highest population density of *C. relictus*, the area is uniquely special in that every year, new species and various newly recorded species are constantly found.

As previously mentioned, Gwangneung Forest has been preserved for over 550 years. It has suffered little human interference and it is considered a climax forest with many loose-flower hornbeams (*Carpinus laxiflora*) and oaks (*Quercus* spp.) with DBH over 40 cm, thus providing an adequate habitat for *C. relictus*. However, the forest is located in a metropolitan area, being surrounded by Namyangju City, Uijeongbu City and Pocheon City, and has therefore suffered an ecological isolation phenomenon resulting from population growth, development and climate change. Moreover, although it is a climax forest in the temperate zone of the Korean Peninsula, the growing stock is of 277 m^2^/ha, i.e. 2.2 times the average of South Korea and with 37.7% of the trees being between 80 and 90 years old, which indicates a declining tendency in the vegetation (Lee et al. 2016). In view of this situation, Gwangneung Forest is considered to be very weak in terms of its capacity to maintain *C. relictus* populations. Therefore, the host plants and their seedlings should be managed so that *C. relictus* can continue to inhabit the forest and old trees before withering should be kept in the forest to feed the larvae. In the long term, it is necessary to find a more stable habitat to replace Gwangneung Forest as a habitat for *C. relictus*.

The worldwide effects of climate change are currently being manifested in various forms. For 106 years (1912–2017), the annual average temperature in Gwangneung Forest rose by 0.18 °C every 10 years and the annual average minimum temperature increased by 0.24 °C every 10 years (Kim et al. 2018). For 36 years (1980–2016) in Pocheon City, the annual mean temperature was 11.7 °C, with the highest and lowest values of 13.2 °C in 2016 and 10.1 °C in 1980, respectively (Korea National Arboretum 2016). The annual mean temperature rises faster every year, indicating that winter is becoming shorter and warmer, which can affect the growth of overwintering insects. The southern distribution limit of *C. relictus* is Gwangneung Forest, located at latitude 38°N, but this limit may move further north as a result of climate change. Therefore, the search for *C. relictus* populations in other areas should be focused on regions for which the species has been recorded in the past, but these areas should also have similar or lower temperatures than that of Gwangneung Forest. Moreover, the selected area should have large and old host plants. Considering these aspects, mountainous areas of Gangwon Province and high-altitude areas with low temperature and little human interference should be considered as strong candidates for the occurrence of *C. relictus* populations.

### Studies on the molecular features of *C. relictus*

Molecular studies on *C. relictus* would allow the inference of the origin and of the systematic relationships among the known populations of the species throughout its distribution area. The Korea National Arboretum performed a COI barcode sequence analysis (Lim et al. 2013) and a mitochondrial sequence analysis (Lim et al. 2017) using Korean *C. relictus*. However, as samples from other countries were difficult to obtain, no comparison among populations was performed. Obtaining living individuals from China was difficult and the specimens deposited in collection were too old for genetic research. In Russia, no quantitative investigation has been conducted, but local populations are known to have been reduced because of deforestation, removal of dead trees and excessive collection. As *C. relictus* is currently an endangered species in Russia and China, the collection and export of samples from these countries is prohibited. Therefore, a joint research project on *C. relictus*, including researchers from South Korea, China and Russia is necessary.

The Korea National Arboretum plans to conduct a study on domestic and foreign *C. relictus* populations with the contribution of research institutes of other countries. Microsatellites, single nucleotide polymorphism markers and Next Generation Sequencing should then be used for genetic linkage mapping and the identification of populations. The knowledge obtained with these techniques can also be used to preserve the genetic uniqueness of the Korean populations by preventing the introgression of artificial foreign populations and to explore functional genes and develop them as genetic resources.

### Minimization of artificial interference and establishment of conservation policies

Before 1980, the Korean populations were widely distributed in the northern part of Gangwon Province (Lee et al. 2018). However, contrary to the protected Gwangneung Forest, most of this area is currently under development or severe deforestation. Although many factors may have contributed to local extinctions of *C. relictus*, small human interferences such as indiscriminate over-catching is believed to greatly affect low-density populations. Therefore, the entire area of occurrence of *C. relictus* and its neighboring areas should be designated as protected areas and public access must be controlled, especially during the emergence period of *C. relictus* adults. Moreover, endangered wildlife poaching should be regulated under stricter laws.

**Figure 11.**
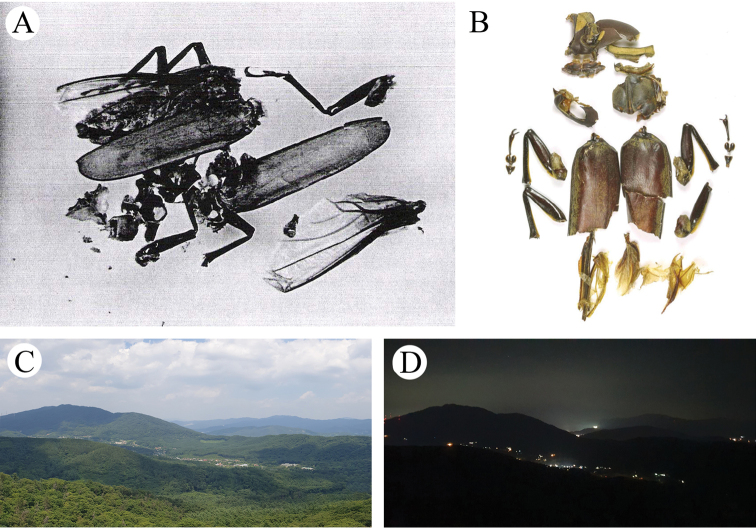
Body fragments of *Callipogon relictus* individuals that had been run over **A** 1978 **B** 2001 and light pollution at night around Gwangneung Forest (**C**).

Byun et al. (2007) mentioned that, in addition to direct interference (e.g., habitat development and illegal poaching), indirect interference also has adverse effects on the maintenance of *C. relictus*. As previously mentioned, Gwangneung Forest is a UNESCO biosphere reserve, which designates core areas and buffer zones to suppress unnecessary development (Lee et al. 2016). However, since the opening of the Korea National Arboretum (1999), the number of restaurants and accommodation facilities has rapidly increased with the increase in the number of tourists and vehicles, threatening the survival of the *C. relictus* population in the area. Two previous case studies reported the behavior of *C. relictus* in the day and night-time: An (2010) mentioned that *C. relictus* is mainly nocturnal as are many other cerambycids; Li et al. (2012) noted that *C. relictus* starts to fly when the temperature rises after 9:00 in Chinese forest conditions and that females lay eggs in suitable places at night. As *C. relictus* adults are attracted to night lights, they are commonly found at the edge of the forest (Byun et al. 2007; Lee et al. 2018), where they are sometimes run over by cars or tourists (Fig. 11A, C) or may be unable to return to their original habitat. Therefore, measures to remove factors that adversely affect the survival of *C. relictus*, such as reducing the brightness of lights from streetlights and restaurants or replacing them with LED lights, should be implemented.

**Figure 12.**
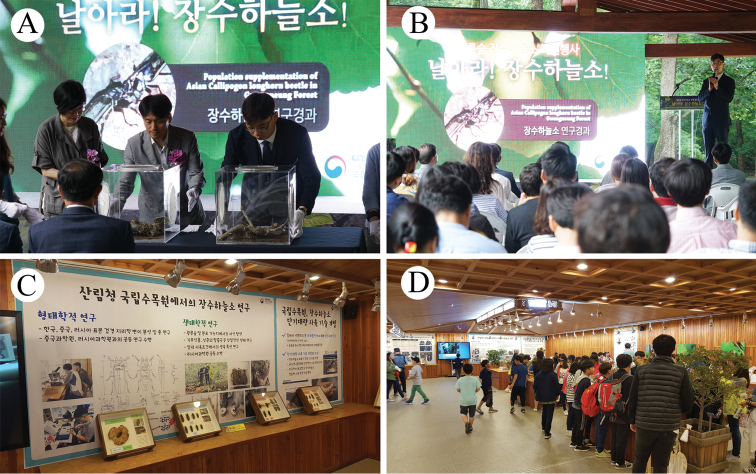
Scientific events, advertisements and educational efforts for the promotion of public awareness regarding the conservation of endangered species such as *Callipogon relictus***A, B** ceremony for the first restoration of *C. relictus* in Gwangneung Forest in 2018 **C, D** special exhibition on *C. relictus* at Korea National Arboretum in 2018.

For the proposed conservation measures to be implemented, a comprehensive conservation policy must be established through continuous communication among the provincial and metropolitan jurisdictions and groups of experts such as those from the Korean National Arboretum. Moreover, the changes in the buffer zone of the Korea National Arboretum must be performed, together with the maintenance of a friendly relationship with local residents of the area, which can be done through raising awareness and education on the importance and value of the species as a future ecotourism resource (Fig. 12A–D).

**Figure 13.**
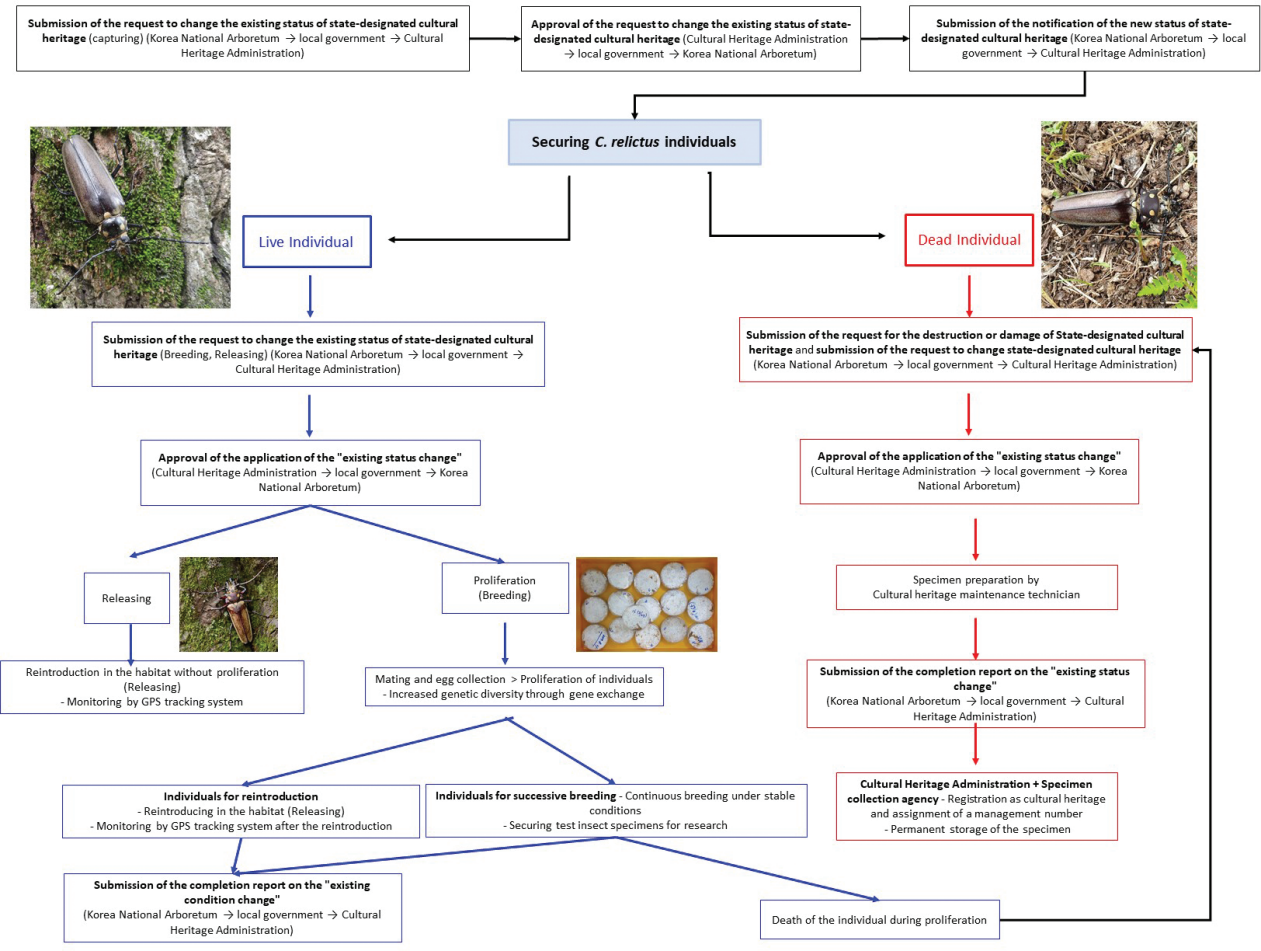
A proposed manual for the conservation of *Callipogon relictus* in Gwangneung Forest.

**Figure 14.**
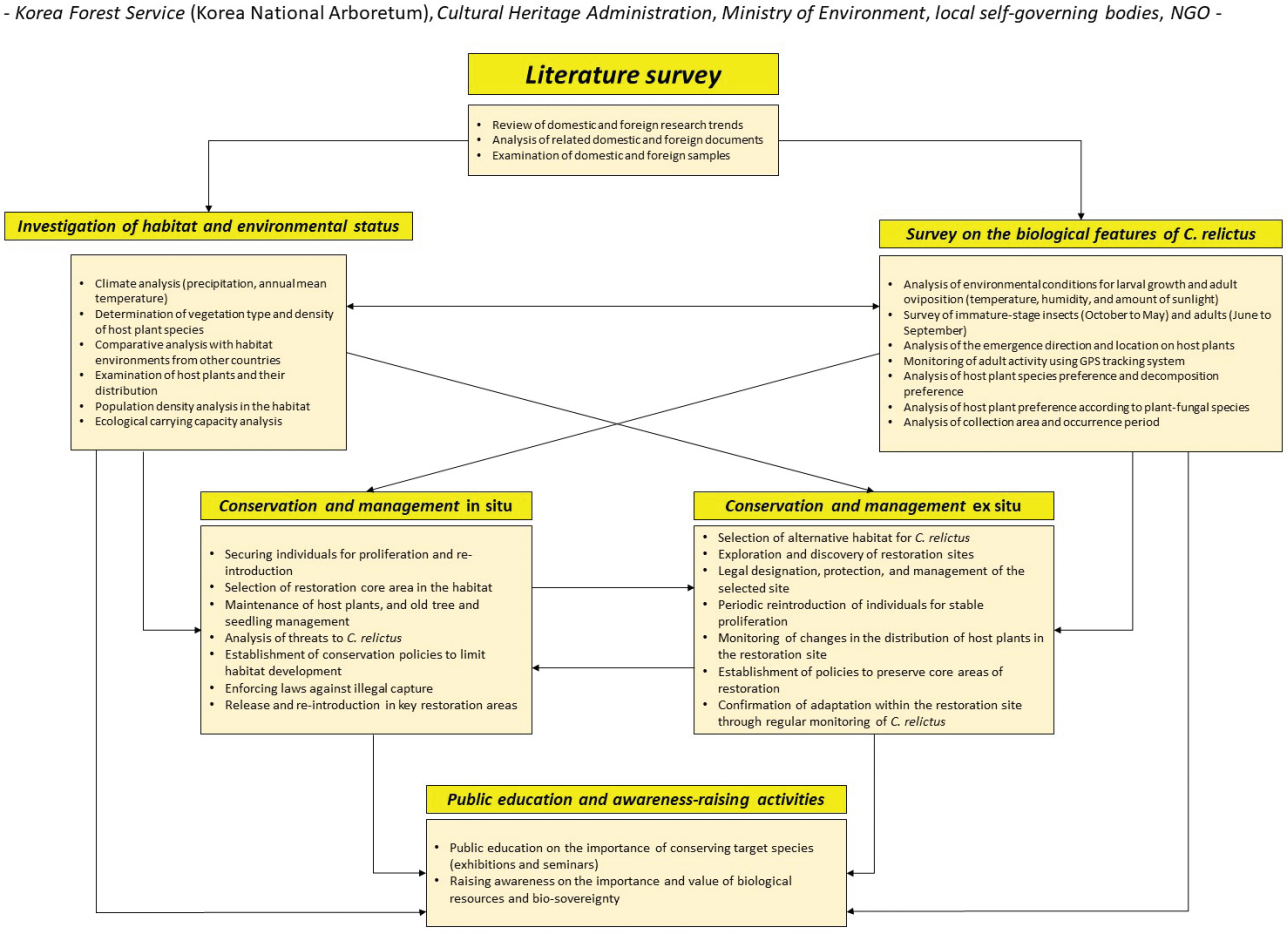
Proposed management measures for the conservation of *Callipogon relictus*.

All actions, including the collection, rearing and releasing of *C. relictus*, require the authorization of the government based on the “Cultural Heritage Protection Act”, which is a process that includes several administrative steps. Herein, we explain the process that should be followed (Fig. 13) and present a management plan for the restoration of *C. relictus* in Gwangneung Forest (Fig. 14). The results of the literature survey show that an investigation on the habitat and environmental status (i.e., biological features) is first needed, followed by restoration and management initiatives involving public education and awareness activities. These systematic management measures could be applied to the restoration of other endangered beetle species and should always be applied together with public education and awareness activities.
